# Laser controlled singlet oxygen generation in mitochondria to promote mitochondrial DNA replication *in vitro*

**DOI:** 10.1038/srep16925

**Published:** 2015-11-18

**Authors:** Xin Zhou, Yupei Wang, Jing Si, Rong Zhou, Lu Gan, Cuixia Di, Yi Xie, Hong Zhang

**Affiliations:** 1 Institute of Modern Physics, Chinese Academy of Sciences, Lanzhou 730000, China; 2 Key laboratory of Heavy Ion Radiation Biology and Medicine Institute of Nuclear Physics, Chinese Academy of Sciences; 3 Key laboratory of Heavy-ion Radiation Medicine of Gansu Province, Lanzhou 730000, China; 4 Graduate School of Chinese Academy of Sciences, Beijing 100039, China

## Abstract

Reports have shown that a certain level of reactive oxygen species (ROS) can promote mitochondrial DNA (mtDNA) replication. However, it is unclear whether it is the mitochondrial ROS that stimulate mtDNA replication and this requires further investigation. Here we employed a photodynamic system to achieve controlled mitochondrial singlet oxygen (^1^O_2_) generation. HeLa cells incubated with 5-aminolevulinic acid (ALA) were exposed to laser irradiation to induce ^1^O_2_ generation within mitochondria. Increased mtDNA copy number was detected after low doses of 630 nm laser light in ALA-treated cells. The stimulated mtDNA replication was directly linked to mitochondrial ^1^O_2_ generation, as verified using specific ROS scavengers. The stimulated mtDNA replication was regulated by mitochondrial transcription factor A (TFAM) and mtDNA polymerase γ. MtDNA control region modifications were induced by ^1^O_2_ generation in mitochondria. A marked increase in 8-Oxoguanine (8-oxoG) level was detected in ALA-treated cells after irradiation. HeLa cell growth stimulation and G1-S cell cycle transition were also observed after laser irradiation in ALA-treated cells. These cellular responses could be due to a second wave of ROS generation detected in mitochondria. In summary, we describe a controllable method of inducing mtDNA replication *in vitro*.

Mitochondria play a crucial role in many aspects of life. The copy number of the mitochondrial genome (mtDNA) varies in response to the physiological environment surrounding the cell^1^. Reactive oxygen species (ROS), which are generated during oxidative phosphorylation, are thought to cause constant oxidatively generated damage to mtDNA. On the other hand, reports indicate that ROS can act as signaling molecules to promote mtDNA replication^2^. However, the exact mechanism by which mitochondria respond to ROS to regulate mtDNA replication remains elusive. Feng *et al*. found that exposing isolated yeast mitochondria to hydrogen peroxide resulted in a simultaneous increase in Ntg1-dependent double strand break (DSB) levels at ori5 and an increase in mtDNA copy number^3^. Their results indicated that mitochondrial ROS regulated mtDNA replication. However, this hypothesis is hard to confirm *in vitro* as it is difficult to selectively trigger ROS generation within mitochondria without affecting the nuclear signaling and cytoplasmic redox state.

^1^O_2_, the lowest excited electronic state of molecular oxygen, is one of the main ROS generated in biological systems. ^1^O_2_ can act directly to oxidatively generate damage and/or as a signaling agent to initiate oxidation reactions^4^,^5^,^6^,^7^. Although ^1^O_2_ is not generated among the main ROS during oxidative phosphorylation, certain levels of ^1^O_2_ can be produced by irradiation of a photosensitizer that is either endogenous or specifically added for this purpose. This process provides an approach for the controlled production of ^1^O_2_ within mitochondria. 5-Aminolevulinic acid (ALA) is the common precursor of tetrapyrrole compounds, and is widely distributed in both plant and animal cells^8^,^9^. In animal cells, ALA is formed from glycine and succinyl CoA by ALA synthase in mitochondria^10^,^11^. Exogenous ALA administration leads to the accumulation of protoporphyrin IX (PpIX) in the mitochondria, which causes direct mitochondrial damage and subsequent cell death after high dose light irradiation via extensive ^1^O_2_ generation^12^,^13^. However, it is unclear whether relatively low doses of mitochondrial ^1^O_2_ could serve as a signaling molecule.

In this study, HeLa cells were incubated with ALA to allow accumulation of PpIX within mitochondria. The generation of ^1^O_2_ within mitochondria, induced by laser irradiation, allowed us to determine whether mitochondrial ROS, such as ^1^O_2_, can act as a signaling molecule to facilitate mtDNA replication. Here we found that mitochondrial ^1^O_2_ promoted mtDNA replication, independently of cellular redox state. Low levels of mitochondrial ^1^O_2_ led to specific displacement loop (D-loop) modifications, which may contribute to the initiation of mtDNA replication. Factors directly involved in the mtDNA replication process such as TFAM and mtDNA polymerase γ regulated the singlet-oxygen-induced mtDNA replication. Moreover, the initial ^1^O_2_ signal may have caused a second wave of ROS generation, which may act as a secondary messenger to regulate cell cycle progression and cell proliferation.

## Results

### Low-dose mitochondrial photodynamic treatment enhanced HeLa cell proliferation

**Figure 1:**
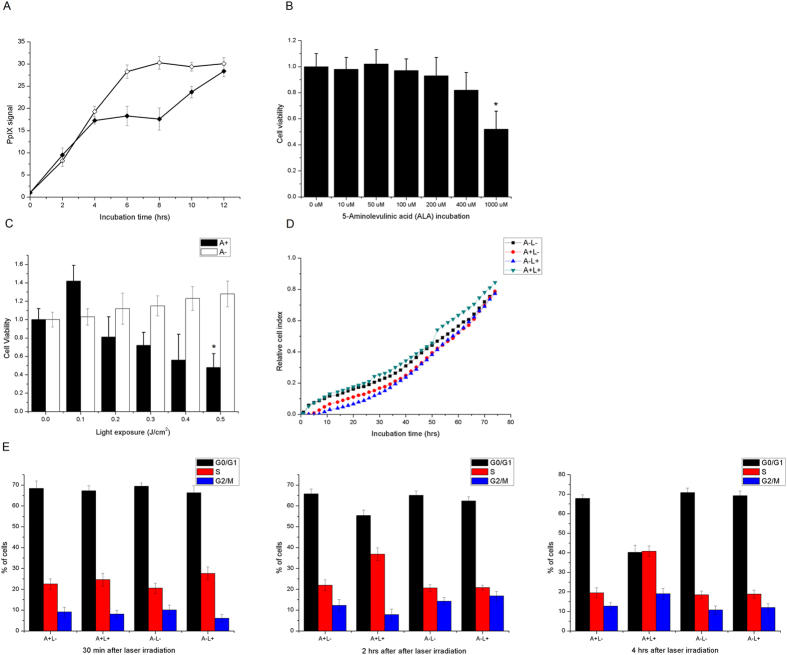
Low dose mitochondrial photodynamic treatment enhanced HeLa cell proliferation. (**A**) Kinetic measurements of PpIX accumulation in HeLa cells following ALA-induction. HeLa cells were incubated with 200 μM ALA for up to 12 h. Open symbols represent experiments with ALA, closed symbols represent experiments with ALA supplemented with CCCP (10 μM) at 4 h and removal of CCCP at 8 h. (**B**) Cell viability was measured by MTT assay 24 h in HeLa cells incubated with 10-1000 μM ALA. (**C**) Cell viability was measured by MTT assay 24 h after laser irradiation in HeLa cells incubated with 200 μM ALA (**D**) Cell growth was monitored by RT-CES System. Laser irradiation was applied at 24 h and 48 h time points. (**E**) FACS analysis to classify each cell cycle. Quantification of every phase by PI staining was performed. The percentage of cells in each phase of the cell cycle was calculated by FACS analysis. Symbols: A: 200 μM ALA incubation; L: 0.1 J/cm^2^ laser irradiation. Each data point represents the mean of three separate experiments; bars are the standard errors. *Statistical significant at p < 0.05 versus control.

We investigated the kinetics of PpIX accumulation in HeLa cells following incubation with 200 uM ALA for up to 12 h by fluorescence measurement at 630 nm. The acquired relative fluorescence values were normalized to the total protein level. The accumulation of PpIX reached a plateau following 8 h of incubation. The addition of 10 μM Carbonyl cyanide m-chlorophenyl hydrazone (CCCP) at 4 h stopped the accumulation of PpIX, while removal of CCCP at 8 h allowed the gradual recovery of PpIX incorporation in the following hours (Fig. 1A).

The HeLa cells were treated with 200 μM ALA for 8 h and then exposed to laser irradiation in order to examine their photosensitivity. Treatment with 10–400 μM ALA showed no toxic effects on HeLa cell viability, while treatment with 1000 μM ALA reduced cell viability to 52% (Fig. 1B). Exposure to the laser alone (from 0.1–0.5 J/cm^2^) did not induce significant cellular inactivation. The lethality of cells treated with 200 μM ALA was dependent on the time of irradiation (Fig. 1C). Interestingly, while most high doses of laser irradiation (from 0.3–0.5 J/cm^2^) showed obvious cytotoxicity in ALA-treated cells, low dose laser irradiation (0.1 J/cm^2^) increased the number of viable cells compared to the control. To further verify this phenomenon, cell growth was measured in the dark for 72 h, following laser irradiation at a dosage of 0.1 J/cm^2^ every 24 h. As shown in Fig. 1D, cells incubated with ALA showed an increase in adherent cells shortly after laser exposure. Furthermore, laser irradiation promoted G1/S checkpoint transition in ALA-treated HeLa cells, as more G1 cells entered into S phase in the following 4 h after laser irradiation (Fig. 1E). No significant changes in cell cycle distribution in ALA incubated cells or laser irradiated only cells were observed. Overall, these data show that low dose mitochondrial photodynamic treatment enhanced HeLa cell proliferation.

### Low dose ^1^O_2_ in mitochondria induction promoted mtDNA replication

**Figure 2:**
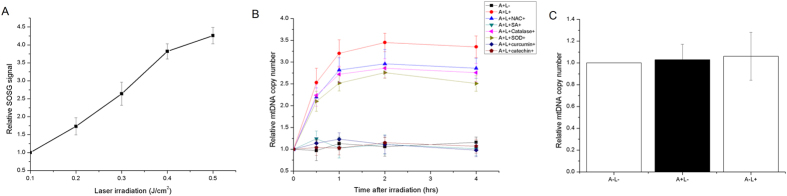
Mitochondrial singlet oxygen promoted mtDNA replication. (**A**) Relative SOSG signal obtained in PpIX solution irradiated by different doses of laser beams. (**B**) Relative mtDNA copy number in ALA-treated HeLa cells after 0.1 J/cm^2^ laser irradiation. (**C**) Relative mtDNA copy number in HeLa cells after after 0.1 J/cm^2^ laser irradiation or 200 μM ALA treatment alone. Symbols: A: 200 μM ALA incubation; L: 0.1 J/cm^2^ laser irradiation; NAC: 10 mM NAC; SA: 10 mM sodium azide. Cat: 200 unit/ml catalase; SOD: 500 unit/ml SOD; Curcumin: 10 mM Curcumin; Catechin: 10 mM catechin. Each data point represents the mean of three separate experiments; bars are the standard errors.

PpIX is an endogenous porphyrin and is synthesized in the mitochondria^14^. It possesses a triplet state that reacts strongly with oxygen to generate ^1^O_2_. To assess whether accumulated PpIX can result in increased ^1^O_2_ generation within mitochondria after laser irradiation, the level of ^1^O_2_ was measured by singlet oxygen sensor green (SOSG) staining. The SOSG signal increased after laser irradiation in a dose-dependent manner (Fig. 2A). No detectable signal for SOSG was obtained in the PpIX solution after laser irradiation in the presence of 10 mM sodium azide (data not shown). A temporary mtDNA copy increase within 4 h after laser irradiation was seen in ALA-treated cells (Fig. 2B). In the controls, neither incubation with 200 μM ALA nor 0.1 J/cm^2^ laser irradiation alone induced mtDNA replication (Fig. 2C). N-acetyl cysteine (NAC), catalase and superoxide dismutase (SOD) were used to eliminate cellular ROS such as hydrogen peroxide (H_2_O_2_) and/or superoxide (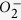), and sodium azide was used to abrogate ^1^O_2_, respectively. None of the ROS scavengers, including NAC (10 mM), SOD (500 unit/ml) and catalase (200 unit/ml) had a significant effect on the promotion of mtDNA replication induced by laser irradiation; while the specific ^1^O_2_ scavenger sodium azide (10 mM) completely abolished the effect induced by laser irradiation (Fig. 2B). As sodium azide is known to quench the triplet state of excited molecules that may be involved in the formation of ^1^O_2_ by energy transfer according to the type II photosensitization mechanism, to further prove the direct involvement of ^1^O_2_ in mtDNA replication, efficient quenchers of ^1^O_2_ were used according to recent reports^15^,^16^. Both catechin (10 mM) and curcumin (10 mM) abolished the stimulation of mtDNA replication. These results further confirmed that ^1^O_2_ stimulated mtDNA replication.

### TFAM and mtDNA polymerase γ mediated ^1^O_2_-induced mtDNA replication

**Figure 3:**
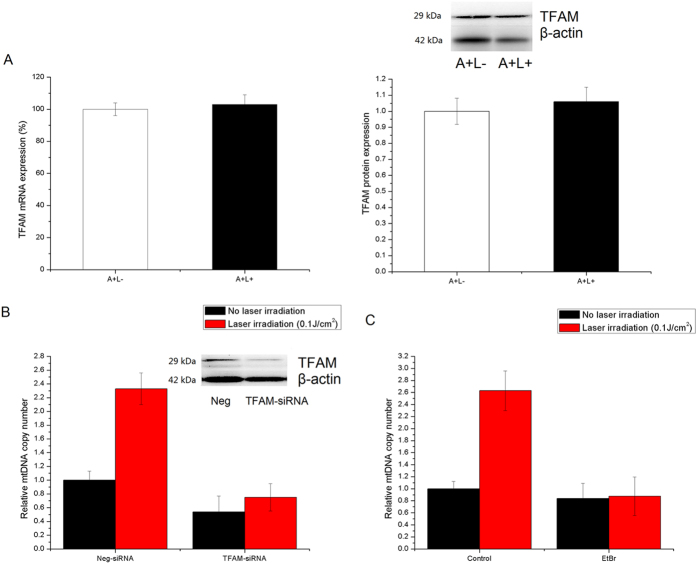
TFAM and mtDNA polymerase γ regulated mtDNA replication. (**A**) The relative TFAM mRNA and protein expression in ALA-treated HeLa cells with/without 0.1 J/cm^2^ laser irradiation. (**B**) siRNA-mediated TFAM knockdown abolished singlet-oxygen-induced mtDNA replication. After HeLa cells were transfected with TFAM specific siRNA (TFAM-siRNA) or Neg-siRNA for 48 hours, 200 μM ALA was added for additional 8 hours following 0.1 J/cm^2^ laser irradiation. The relative mtDNA copy number was then measured 0.5 hours after laser irradiation. (**C**) Inhibition of mtDNA polymerase γ by addition of 100 ng/ml EtBr could abrogate the increase of mtDNA copy number stimulated by singlet oxygen in ALA-treated cells. Each data point represents the mean of three separate experiments; bars are the standard errors.

TFAM is an essential component of nucleoid structure and is necessary for transcription and replication initiation of mtDNA. No significant changes in mRNA or protein expression of TFAM were detected in ALA-treated cells at the point of increased mtDNA copy number (30 min after laser irradiation) (Fig. 3A). However, the stimulatory role of ^1^O_2_ on mtDNA copy number was partially diminished by TFAM knock down with gene-specific siRNA (Fig. 3B), indicative of the mediation of TFAM in ^1^O_2_-induced mtDNA replication.

As mtDNA polymerase γ is directly involved in the mtDNA replication process, ethidium bromide (EtBr), a specific mtDNA polymerase inhibitor was used to determine whether inhibition of mtDNA polymerase could block the mtDNA replication stimulated by mitochondrial ^1^O_2_^17^. The addition of EtBr (100 ng/ml) 1 h prior to laser irradiation significantly abrogated the increase in mtDNA copy number. These results suggest that mitochondrial ^1^O_2_-induced mtDNA replication is tightly controlled by the activity of mtDNA polymerase γ.

### ^1^O_2_ in mitochondria induced D-loop modification and a second wave of ROS generation

**Figure 4:**
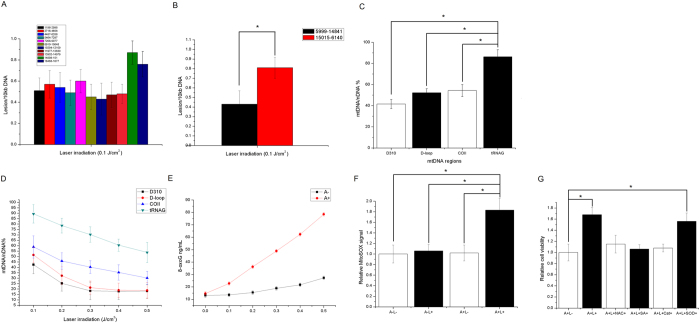
Singlet oxygen induced regional mtDNA damage and second wave of ROS generation. (**A**) Quantification of mtDNA damage per 10 kb DNA by SLR rt-PCR amplification of total DNA isolated from ALA-treated HeLa cells exposed to 0.1 J/cm^2^ laser irradiation. Region 14898-151 and 16488-1677 exhibited significant increased mtDNA damage, overlapping the control region of human mitochondrial genome. (**B**) Quantitative measurement of mtDNA damage using Long-run quantitative PCR. Representation of the DNA lesions of the 8.9 kb and 9.4 kb mtDNA fragments. Lesion frequencies of treated samples were calculated per amplicon size and expressed per 10 kb of mitochondrial genome. (**C**) mtDNA-damage profile of specific mtDNA regions after exposure to 0.1 J/cm^2^ laser irradiation in ALA-treated HeLa cells. (**D**) Dose-dependent relative mtDNA damage in four mtDNA regions of ALA-treated HeLa cells. (**E**) Isolated mtDNA from HeLa cells was analyzed for 8-oxoG accumulation using an ELISA assay. Oxidized lesions were quantified according to a standard curve generated using known amounts of 8-oxoG. (**F**) Mitochondrial oxidative stress was monitored by the mean MitoSOX fluorescence using plate reader where HeLa cells were incubated with 200 μM ALA, follow by laser irradiation or not. (**G**) Relative cell index was measured by MTT assay 24 h after laser irradiation with or without ROS scavenger. Symbols: A: 200 μM ALA incubation; L: 0.1 J/cm^2^ laser irradiation; NAC: 10 mM NAC; SA: 10 mM sodium azide; Cat: 200 unit/ml catalase; SOD: 500 unit/ml SOD. Error bars represent the SD, each done in at least triplicate. *Statistical significant at p < 0.05.

The D-loop is a 1.1 kb cis-regulatory region which is important in replication and transcription of mtDNA^18^. Feng *et al*. found that mtDNA is oxidatively modified at active replication origins such as ori5 in response to increased ROS^3^. To assess whether there was a specific modification in the D-loop region in HeLa cells with increased mitochondrial ROS, a semi-long run real-time PCR (SLR rt-PCR) analysis was used to screen the regional mtDNA damage in ALA-treated cells after laser irradiation. As shown in Fig. 4A, the SLR rt-PCR analysis revealed a similar lesion rate in all the encoding regions from position 1196 to 14979. The two regions that overlapped the non-coding region of mtDNA (4898-151 and 16488-1677, respectively) showed increased mtDNA lesions compared to the remaining regions. The selective modification of the D-loop region was further confirmed by Long-run quantitative PCR, as the mtDNA lesions of the 9.4 kb fragment harboring the D-loop region were much higher than the 8.4 kb amplicon that did not contain a D-loop region after laser irradiation in ALA-treated cells (Fig. 4B). As the D310 region lies in the conserved sequence block in the D-loop that is involved in mtDNA replication and transcription^18^, we further examined whether there were specific modifications in the D310 region after exposure to laser irradiation. To quantify ^1^O_2_-induced damage in tRNAG, COII, D-loop and D310 a method adapted from Manbo *et al*.^19^ was used. The results showed that the D310, D-loop and COII region were more sensitive to ^1^O_2_ compared with the coding region tRNAG in ALA-treated cells after 0.1 J/cm^2^ laser irradiation (Fig. 4C). Moreover, increasing the laser irradiation dosage resulted in preferential damage in the D310 region, followed by the D-loop and COII (Fig. 4D). There was significantly greater mtDNA damage in the D310 region compared to tRNAG in cells after 0.5 J/cm^2^ laser irradiation. These results confirmed that there was specific modification in the D-loop region with the generation of mitochondrial ^1^O_2_, which may contribute to the initiation of mtDNA replication.

It is well documented that 8-oxoG is the main ^1^O_2_ oxidation product of nuclear DNA in cells, which may also apply to mtDNA. Moreover, the D310 region contains sequences highly enriched with guanine (GGGGGGGAGGGGG), which is part of the conserved sequence block II and is involved in the formation of a persistent RNA-DNA hybrid that leads to the initiation of mtDNA heavy-strand replication^20^. To determine whether ^1^O_2_ generated by PpIX photosensitization induced 8-oxoG in mtDNA, the overall 8-oxoG level in mitochondria was then quantified by ELISA. 8-oxoG lesions in mtDNA increased with laser irradiation dosage in ALA-treated cells. A slight increase in 8-oxoG lesions in control cells exposed to laser irradiation was seen, but these were significantly fewer compared to those in ALA-treated cells (Fig. 4E).

MitoSOX is a Mitochondrial-targeted dihydroethidium, which is suitable for the detection of mitochondrial 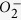. In addition, researchers have found that MitoSOX also reacts with other oxidants (cytochrome *c*, peroxidase and H_2_O_2_)^21^,^22^, thus the overall fluorescence recorded may reflect the general oxidative stress within mitochondria. We found increased mitochondrial oxidative stress, as detected by MitoSOX, compared to the control in ALA-treated cells after laser exposure (Fig. 4F). The laser-induced stimulation of growth in ALA-cells was abolished by both NAC and sodium azide addition, as measured by the MTT assay 24 h after irradiation (Fig. 4G). The addition of catalase, but not SOD, abrogated this effect, suggesting that H_2_O_2_ participated in the progression of cell growth stimulation.

## Discussion

In photodynamic therapy (PDT), particular attention has been focused on the ability of ^1^O_2_ to trigger cell death, and it is unclear whether ^1^O_2_ can act as a signaling agent to regulate certain biological events, such as mitochondrial biogenesis. To our knowledge, this is the first study to investigate the functional role of mitochondrial ^1^O_2_ in mtDNA replication.

ALA has been shown to have toxic effect on cells, especially on the normal function of mitochondria. Janice *et al*. found that incubation with 20 mM ALA led to significant mtDNA damage in the Simian virus 40 (SV40)-transformed human fibroblast cell line, GM00637E^23^. Jihane *et al*. reported that treatment with 1-300 μM ALA for 24 h had no effect on cell viability^24^. In the same study, it was found that 300 μM ALA reduced mtDNA content by 50%. These studies show that ALA is directly implicated in mitochondrial toxicity in a concentration-dependent and cell-specific manner. In our study, 200 μM ALA was used to treat HeLa cells, and this treatment alone showed neither significant cell growth inhibition nor proliferation. This may have been due to the relatively low dosage of ALA used. In another study using HeLa cells, 1 mM ALA combined with laser exposure led to a significant proliferative effect^25^. These results indicate that the effects of ALA on cells and mitochondria may differ in a dose-dependent manner. It was previously reported that ALA-treated HeLa cells exposed to 0.14 J laser irradiation showed an enhanced proliferation rate compared to the control^26^. Consistent with their findings, we also observed that low dose laser beams promoted cellular proliferation in HeLa cells incubated with ALA. It should be noted that reports have shown that low dose laser irradiation alone can promote cell proliferation^27^,^28^,^29^. However, no significant cell proliferation was observed in our experiment using laser irradiation alone (up to 0.5 J/cm^2^). This may be due to the fact that even the highest dose of laser irradiation used was below the lower limit to promote cell proliferation seen in other studies^28^.

^1^O_2_ generation has been reported to occur in various biological processes as indicated by low-level chemiluminescence^30^,^31^,^32^,^33^. There is evidence to show that ^1^O_2_ may be involved in physiological and pathophysiological events in tissues. Moreover, a study has shown that ^1^O_2_ can be produced enzymatically even in the dark^34^. ^1^O_2_ generated by PpIX photo-activation reacts primarily in the mitochondrial matrix, as the key enzymes of PpIX synthesis are localized in this compartment^35^,^36^. Thus, mtDNA may be one of the main targets of ^1^O_2_ attack, due to its localization within the mitochondrial matrix. However, although 5-ALA-mediated photodynamic effects on mitochondrial biogenesis have been reported^14^,^37^, there are currently no data on its effect on mtDNA. To our knowledge, these are the first results of mitochondrial ^1^O_2_-induced mtDNA modifications. Our data show that the mtDNA control region was preferentially damaged by mitochondrial ^1^O_2_.

Mitochondria are the major source of intracellular ROS. The proximity of mtDNA to the respiratory chain and oxidative phosphorylation results in an increased likelihood of ROS-induced mtDNA damage. However, recent experimental evidence has called into question mitochondrial ROS as a damaging agent^38^. It is thought that ROS can function as signaling molecules to facilitate adaptation to stress in physiological situations^39^. Mild cellular damage was suggested to increase the rate of the synthesis-degradation cycle of mtDNA^40^. Certain low levels of ROS can promote mtDNA replication instead of degradation in mitochondria isolated from yeast^3^. Our previous study also showed that low dose X-ray irradiation could induce a transient increase in mtDNA copy number^41^. However, *in vitro* experiments to determine whether mitochondrial ROS can serve as signaling molecules to promote mtDNA replication, rather than induce mtDNA damage in mammalian cells are still lacking. Here we showed that mitochondrial ^1^O_2_ generated by controllable laser irradiation stimulated mtDNA replication. The initial increase in mtDNA copy number 30 min after irradiation was caused by ^1^O_2_ stimulation to a large extent, as most ROS scavengers showed no significant effect on the initial increase, while sodium azide did affect this increase. Our data also showed that mtDNA replication was at least partially regulated by ROS, as there was a general decrease in mtDNA copy number following the addition of NAC 2 h after irradiation (Fig. 2). How exactly does ^1^O_2_ stimulate initial mtDNA replication? This question remains unsolved. Feng *et al*. found increased DSB levels at *ori5* in H_2_O_2_-treated mitochondria, which can lead to the initiation of mtDNA replication. Our previous study also found that mtDNA damage induced by X-rays was mostly funneled to the control region via a charge transport mechanism^42^. Interestingly, both loci which are the active replication origins (*ori5* in yeast and D-loop in human cells) contain sequences highly enriched with guanine (GGGAGGGGGTGGGTG in *ori5* and GGGGGGGAGGGGG in the control region). While ^1^O_2_ specifically reacts with biomolecules exhibiting double bonds rich in electrons, such as guanine^43^, the specific mtDNA modification at the control region could possibly arise from selective oxidation of the guanine base by ^1^O_2_ (type II reaction)^44^,^45^,^46^. Indeed, we found increased 8-oxoG levels in mtDNA during ^1^O_2_ induction. However, more detailed studies are necessary in the future to determine whether ^1^O_2_ in mitochondria results in specific guanine modifications in the D-loop region. Here we postulated that selective modification of the mtDNA control region induced by ^1^O_2_ could initiate mtDNA replication, which resembles H_2_O_2_-induced mtDNA replication in yeast mitochondria^3^.

Here we showed that mitochondrial ^1^O_2_ could act as a signaling molecule to initiate mtDNA replication. However, the latent biological events, such as G1-S transition and cell proliferation, were not directly mediated by mitochondrial ^1^O_2_. ^1^O_2_ has a life-time of less than 0.05 μs in cells with a diffuse length of less than 0.02 μm^47^, so short that its intracellular targets are the sites where it is generated^48^. Therefore, it is unlikely that the biological events outside the mitochondrial matrix, such as cell proliferation and G1-S transition, could be due to the direct action of mitochondrial ^1^O_2_. This could explain the latency between mtDNA replication and cell proliferation (0.5 h versus >2 h).

An early study showed that singlet oxygen induced by hypericin could inhibit succinoxidase in isolated mitochondria^49^. This finding indicated that ^1^O_2_ may perturb the mitochondrial electron transport chain (ETC) by succinoxidase inhibition, thus leading to possible further generation of excess ROS. The transient production of H_2_O_2_ is considered an intracellular signal for cell growth and transformation^50^. Mitochondria-derived ROS generation, especially H_2_O_2_, could act as the mediator between mitochondrial ^1^O_2_ and nuclear signaling. To determine whether the mitochondria-originated ROS contribute to the promotion of cell proliferation, ROS scavengers were added to ALA-treated cells before laser irradiation. Addition of the ROS scavengers, NAC and catalase, abolished the promotion of cell proliferation, indicating that secondary ROS, such as H_2_O_2_ could serve as a messenger from mitochondria to the nuclear region. Although the addition of SOD did not affect cell growth, we found increased mitochondrial oxidative stress in ALA-treated cells after irradiation, as indicated by MitoSOX red fluorescence. The increased signal of MitSOX indicated elevated mitochondrial 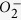 and other oxidants after irradiation. We postulated that the seemingly ineffective addition of SOD may be due to the fact that mitochondrial 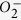 can be efficiently converted to H_2_O_2_, and the lower reactivity of H_2_O_2_ results in longer lifetime and much greater diffusion distance to serve as the secondary ROS messenger.

In addition, a study showed that ^1^O_2_ can react with ascorbate to produce H_2_O_2_^51^. These findings suggest the importance of H_2_O_2_ in the crosstalk between mitochondria and the nucleus.

In summary, we demonstrated that mtDNA replication can be stimulated by a photodynamic approach in mammalian cells. This is the first evidence to show that mitochondrial ^1^O_2_ promotes mtDNA replication. These results confirm that certain low levels of ROS can act as signal molecules rather than toxic agents. The mtDNA replication, triggered by mitochondrial ^1^O_2_, which is independent of cellular redox state, indicates the semi-autonomous feature of mitochondria.

## Material and Methods

### Cell cultures

Human cervix cancer cells HeLa were cultured at 37 °C under humidified 95% air/5% CO_2_ in DMEM supplemented with penicillin (100 units/ml), streptomycin (100 mg/ml), and 10% fetal calf serum. Cell viability was measured using 3-(4,5-dimethyl-thiazol-2-yl)-2,5-diphenyl-2 H-tetrazolium bromide (MTT) assay 24 hours after laser irradiation. Cell viability was expressed as a percentage of control cells. Each experiment was carried out in triplicate.

### Exposure of the cells to laser

The cells were exposed to the red light generated by the He-Ne laser (630 nm, corresponding to the maximum of PpIX absorbance in Q band) (Institute of Modern Physics, Chinese Academy of Sciences). Irradiation times were adjusted to deposit energies of 0.1 to 0.5 J/cm^2^. Each experiment was carried out in triplicate.

### PpIX Kinetics measurement

HeLa cells were harvested in an exponential growth phase and incubated at 37 °C in 96-well plate in 0.2 ml DMEM medium for 6 h. Two sets of measurement were performed, one with CCCP and one without. The medium of the cell-seeded dishes was then replaced with medium containing 200 μM ALA. Untreated cells were used as the drug-free control. Cells were treated with the drug-containing medium under dark conditions for 0–12 hours. The cells were then washed twice with PBS and the fluorescence was measured with a plate reader (Tecan infinite 200 M). The excitation wavelength was 405 nm and the emission wavelength was 631 nm for kinetic measurement. The relative amounts of PpIX were normalized to the total protein level extracted from parallel HeLa cells, as quantified by standard BCA protocol.

### ^1^O_2_ detection in cell free system

^1^O_2_ sensor green reagent (SOSG) (Invitrogen, USA) was used as the ^1^O_2_-tracking agent. 100 μL of 400 μM PpIX (Sigma) solution was directly mixed with 100 μL of 200 μM SOSG in PBS to study the effect of laser irradiation on ^1^O_2_ generation. The fluorescence measurement of SOSG was performed using a plate reader (Tecan infinite 200 M) at an excitation/emission wavelength of 505/525 nm accordingly.

### Cell cycle analysis

For the cell cycle assay, cells were harvested at indicated time points after irradiation and fixed in 70% ethanol at 4 °C overnight. After washing twice with PBS, the cells were incubated with 30 μg/mL propidium iodide (PI), 0.2 mg/mL RNase A, and 0.2% Triton X-100 (Sigma) at 37 °C for 30 minmin. The cells were then analyzed by flow cytometry (BD Biosciences).

### Cell Growth

Real-time monitoring of cell growth was carried out as our previous study^52^. Briefly, cell growth was determined by real-time monitoring of adherent cells by the RT-CES System (ACEA Biosciences). 5000 cells were seeded in each 360 μL well with 200 μL medium 14 hours before measurement to ensure their adherence to the plate recorder. The medium was replaced every 24 hours. Proliferation of HeLa cells was recorded every 2 hours for 72 hours.

### Mitochondrial ROS measurement

For mitochondrial ROS measurement, MitoSOX was used to quantify 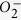 level and other oxidant in mitochondria. HeLa cells were harvested in an exponential growth phase and incubated at 37 °C in 96-well plate in 300 μl DMEM medium for 6 h. At indicated time points after treatments, cells were washed twice with PBS, then treated with 1 μM MitoSOX Red (Life Technologies, USA) in serum free DMED for 20 min at 37 °C in a 5% CO_2_ incubator. After treatment, the cells were washed twice with PBS and fluorescent intensity was measured using excitation at 510 nm and emission at 580 nm.

### DNA isolation and purification

Total DNA was purified using DNA Blood and Tissue Kit (Tiangen) from cells with/without ionizing radiation, respectively, and DNA quantity and purity was determined by spectrometric analysis. The isolated DNA showed a high purity (A260/A280 > 1.8) and was stored at 4 °C according to standard procedures.

### Real time RT-PCR

Total RNA was isolated using RNA extraction kit (Tiangen). First strand cDNA was synthesized according to the manufacturer’s protocol. Real-time PCR was performed in the FTC-3000 qPCR system (FUNGLYN, Canada) using SYBR Green PCR master mix (Takara, Japan). The primers were used as shown below: TFAM (5′-GGAGGCAAAGGATGATTCGG-3′ and 5′-TCGTCCAACTTCAGCCATCT-3′) and β-actin (5′-ATGGTGGAATGGGTCAGAA-3′ and 5′-CTTTTCACGGTTGGCCTTAG-3′). β-actin was used as internal reference. The PCR conditions were as follow: The cycling conditions include 40 cycles of 10 s 95 °C, 10 s 55 °C, and 60 s 72 °C. Each sample was assayed in quadruplicate, fluorescence was continuously monitored versus cycle numbers and threshold cycle (Ct) values were calculated automatically. Relative mRNA quantification was calculated by using the arithmetic formula 2−∆∆CT method.

### Western blotting

All samples were mixed with loading buffer and subjected to 10% SDS–polyacrylamide gel electrophoresis. Each lane was loaded with 50–100 μg of equal amounts of protein. All samples were then transferred onto PVDF membranes and blocked with 5% milk in Tris-buffered saline–Tween 20 (TBST) buffer for 1 h at room temperature. The membranes were subsequently exposed to first antibody against TFAM (29 kDa) and β-actin (42 kDa) (Abcam Inc, USA) at the dilution of 1:1,000 in 5% milk in TBST for overnight at 4 °C, and then membranes were washed and incubated with secondary antibody. Antibody binding was detected using enhanced chemiluminescence (BioRad). The results were quantified by densitometry using Image J software.

### Semi-long run rt-PCR

The semi-long run rt-PCR was carried out as our previous study^42^. Briefly, The SLR rt-PCR amplifications were conducted in the FTC-3000 qPCR system (FUNGLYN,), and the amplification was monitored and analyzed by measuring the intercalation of the fluorescent dye to double-stranded DNA supplied by the MightyAMP ver.2 kit (Takara). The mtDNA regions, primers and their PCR efficiency for rt-PCR are displayed in supplement Table1.

The PCR conditions for the different fragments were optimized to achieve similar amplification efficiencies required to compare different amplicons. The product specificity was monitored by melting curve analysis and sequenced by Sangon, Shanghai for identification (data not shown). The reaction mix (total volume V = 20 μl) consists of 1 × MightyAMP real time mix (Takara), 500 nM each forward and reverse primer and the equivalent quantities of template DNA (10 ng of total DNA). The cycling conditions include a pre-incubation phase of 1 min at 95 °C followed by 40 cycles of 10 s 95 °C, 10 s 55 °C, and 60 s 72 °C. Each sample was assayed in quadruplicate, fluorescence was continuously monitored versus cycle numbers and threshold cycle (Ct) values were calculated automatically.

Data analysis is based on the measurement of the Ct. Isolated total DNA from untreated sample was taken as reference. For each of the 11 mtDNA regions the difference in the Ct was used as a measure of the relative mtDNA damage with the 2−ΔCt method in correlation to the amplification size of the long fragment^53^. The Ct of irradiated mtDNA versus the Ct of non-irradiated mtDNA was directly employed to represent the level of mtDNA damage in each region. The DNA damage was calculated as lesion per 10 kb DNA of each mtDNA region by including the size of the respective long fragment and displayed as average of at least three independent experiments.


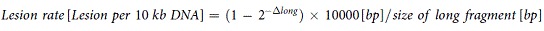


### MtDNA copy number quantification

Total genomic DNA of 50 ng were used for mtDNA and nuclear DNA markers, respectively, in a 20 μl reaction containing 1X SYBR Premix Ex Taq II (TaKaRa, Japan) and 200 nmol/l each primer. The sequence information regarding the primers was as follow: ND1: CACCCAAGAACAGGGTTTGT and TGGCCATGGGTATGTTGTTAA; actin: CAAAACCTAACTTGCGCAGA and TTTTAGGATGGCAAGGGACT. Triplicate reactions were performed for each marker in 50 μl tubes using a three-step amplification program of 40 cycles of 95 °C for 5 s, 55 °C for 10 s and 72 °C for 10 s. Standard curves were generated in each reaction using serial dilutions of untreated genomic DNA. Relative mtDNA copy number was calculated as the ratio of the amplification amount obtained with mtDNA markers versus β-actin for each sample and plotted normalized to the control group.

### Quantitative real-time PCR for different short mtDNA regions

This method is adapted from Manbo *et al*.^19^. Briefly, The PCR amplifications were conducted as described above. Four mtDNA regions including mtDNA regions cytochrome c oxidase (COII), tRNA coding for glycine (tRNA_G_), D-loop (401–490), and D310, and one nuclear DNA β-actin were used (supplement table 1). mtDNA/nDNA ratios were calculated by dividing the mtDNA signal for each gene by the β-actin signal and expressing the ratio as a percentage of the untreated control set at 100%.

### Short interfering RNA (siRNA) transfection

HeLa cells were transfected with gene specific siRNA targeting TFAM (Santa Cruz Biotechnology, USA) for 48 hours using LipofectamineTM2000 transfection agent in serum-free medium following manufacturer’s protocol. The cells transfected with scrambled siRNA acted as a non-silencing control.

### Measurement of 8-oxoG

Mitochondrial DNA was extracted using the mitochondrial DNA isolation kit (Abcam, USA). A commercially available ELISA kit from abcam was used to determine levels of 8-hydroxy-2′-deoxyguanosine (8-oxoG) in isolated DNA. The assays were performed according to the manufacturer’s instructions. The 8-oxoG standard (0.01–30 ng/mL) or 5-μg DNA was incubated with an antimouse IgG-coated plate with a tracer consisting of an 8-oxoG–enzyme conjugate. The assay was normalized by an equal amount of DNA used for each sample. Addition of a substrate to replicate samples was followed by measurement of absorbance at 412 nm. Standard curves were calculated for all reactions with serial dilutions of 8-oxoG standard to calculate reaction efficiency. Samples were assayed in triplicate.

### Statistical analysis

Statistical analysis was performed on the means of the data obtained from at least three independent experiments. Data are presented as means ± SD. Student’s t-test program in Microsoft Excel was used to detect statistical significance. p < 0.05 was considered to be statistically significant.

## Additional Information

**How to cite this article**: Zhou, X. *et al*. Laser controlled singlet oxygen generation in mitochondria to promote mitochondrial DNA replication *in vitro*. *Sci. Rep*. **5**, 16925; doi: 10.1038/srep16925 (2015).

## Supplementary Material

Supplementary Information
