# Supplementary material for: Laser controlled singlet oxygen generation in mitochondria to promote mitochondrial DNA replication in vitro
Source: Sci Rep. 2015 Nov 18;5:16925. doi: 10.1038/srep16925 (PMC4649627; doi:10.1038/srep16925)
Supplement: Supplementary Information [file srep16925-s1.doc]

**Laser controlled singlet oxygen generation in mitochondria to promote mitochondrial DNA replication in vitro**

**Xin Zhou^1,2,3#^, Yupei Wang^1,2,3^, Jing Si^1,2,3^, Rong Zhou^1,2,3^, Lu Gan^1,2,3,4^, Cuixia Di^1,2,3^, Yi Xie^1,2,3^, Hong Zhang^1,2,3^***

1 Institute of Modern Physics, Chinese Academy of Sciences, Lanzhou 730000, China

2 Key laboratory of Heavy Ion Radiation Biology and Medicine Institute of Nuclear Physics, Chinese Academy of Sciences

3 Key laboratory of Heavy-ion Radiation Medicine of Gansu Province, Lanzhou 730000, China

4 Graduate School of Chinese Academy of Sciences, Beijing 100039, China

.

Correspondence to:

Hong Zhang, Institute of Modern Physics, Chinese Academy of Sciences, Lanzhou 730000, China, email: [Zhangh@impcas.ac.cn](mailto:Zhangh@impcas.ac.cn)

Table 1

| MtDNA region | Primer sequences | Product size（bp） | PCR efficiency (%) |
| --- | --- | --- | --- |
| 5464-7287 | F: TTACCACGCTACTCCTACCTAT  R: GAGAAATGAATGAGCCTACAGAT | 1823 | 83.2 |
| 4487-6286 | F: ACCCGTCATCTACTCTACCATC  R: ACTGTTCAACCTGTTCCTGCT | 1799 | 79.4 |
| 7266-9077 | F: TCTGTAGGCTCATTCATTTCTC  R: ATGGTTGATATTGCTAGGGTG | 1811 | 88 |
| 8910-10648 | F : CTTACCACAAGGCACACCTACA  R:ATGGACCACAAGGCACACCTACA | 1738 | 85.1 |
| 10294-12107 | F:TACAAACAACTAACCTGCCACT  R: GTTGAGGGATAGGAGGAGAAT | 1815 | 83.6 |
| 11977-13830 | F:CTCCCTCTACATATTTACCACAAC  R:AAGTCCTAGGAAAGTGACAGCGA | 1853 | 87.8 |
| 13902-14979 | F: CTCCAACATACTCGGATTCTAC  R: ATTCAGCCATAATTTACGTCTC | 1047 | 92 |
| 14898-151 | F: TAGCCATGCACTACTCACCAGA  R:GGATGAGGCAGGAATCAAAGAC | 1849 | 84.8 |
| 16488-1677 | F: CTGTATCCGACATCTGGTTCCT  R : GTTTAGCTCAGAGCGGTCAAGT | 1785 | 81.1 |
| 1196-2998 | F: AGAGGAGCCTGTTCTGTAATCG  R: ATCCAACATCGAGGTCGTAAA | 1802 | 84.7 |
| 2716-4658 | F: GACGAGAAGACCCTATGGAGC  R: TTGCGTGAGGAAATACTTGATGG | 1942 | 78.7 |
| 14620-14841 | F:CCCCACAAACCCCAT TACTAAACCCA  R:TTTCATCATGCGGAGATGTTGGATGG | 222 | 99.5 |
| 401-490 | F: CACAGACATCATAACAAAAAATTTCC  R: GGTGTTAGGGTTCTTTGTTTTTGG | 90 | 99.9 |
| 3459-3562 | F: ACGCCATAAAACTCTTCACCAAAG  R: TAGTAGAAGAGCGATGGTGAGAGCTA | 104 | 98.9 |
| 8456-8557 | F:ACAAACTACCACCTACCTCCC  R:CAATGAATGAAGCGAACAGAT | 102 | 99.9 |
| 15015-6140 | F:ATTCTTTATCTGCCTCTTCCTAC  R: TCGTTGACCTCGTCTGTTATG | 9417 | n/a |
| 5999-14841 | F:TCTAAGCCTCCTTATTCGAGCCGA  R:TTTCATCATGCGGAGATGTTGGATGG | 8843 | n/a |
| D-loop | F: TATCTTTTGGCGGTATGCACTTTTAACAGT  R: TGATGAGATTAGTAGTATGGGAGTGG | 90 | 99.6 |
| D310 | F:CACACAGACATCATAACAAAAAATTTCC  R: GGTGTTAGGGTTCTTTGTTTTTGG | 110 | 98.5 |
| COII | F: CCCCACATTAGGCTTAAAAACAGAT  R: ACCGCTACACGACCGGGGGTATA | 81 | 99.2 |
| tRNA_G_ | F: GCCACCTATCACACCCCATC  R: TACCCTTCCCGTACTAATTAATCCC | 101 | 99.9 |
| β-Actin | F: TCACCCACACTGTGCCCATCTACGA  R: CAGCGGAACCGCTCATTGCCAATGG | 295 | 99.3 |
